# Retrospective Analysis of Training and Its Response in Marathon Finishers Based on Fitness App Data

**DOI:** 10.3389/fphys.2021.669884

**Published:** 2021-05-21

**Authors:** Markus Zrenner, Christian Heyde, Burkhard Duemler, Solms Dykman, Kai Roecker, Bjoern M. Eskofier

**Affiliations:** ^1^Machine Learning and Data Analytics Lab, Department Artificial Intelligence in Biomedical Engineering, University of Erlangen-Nürnberg (FAU), Erlangen, Germany; ^2^Adidas AG, Future Sport Science, Herzogenaurach, Germany; ^3^Adidas AG, Technology & Innovation, Herzogenaurach, Germany; ^4^Runtastic GmbH, Pasching, Austria; ^5^Institute for Applied Public Health and Exercise Medicine, Furtwangen University (HFU), Furtwangen, Germany

**Keywords:** marathon training, big data, wearables, training response, exercise physiology

## Abstract

**Objective:** Finishing a marathon requires to prepare for a 42.2 km run. Current literature describes which training characteristics are related to marathon performance. However, which training is most effective in terms of a performance improvement remains unclear.

**Methods:** We conducted a retrospective analysis of training responses during a 16 weeks training period prior to an absolved marathon. The analysis was performed on unsupervised fitness app data (Runtastic) from 6,771 marathon finishers. Differences in training volume and intensity between three response and three marathon performance groups were analyzed. Training response was quantified by the improvement of the velocity of 10 km runs Δ*v*_10_ between the first and last 4 weeks of the training period. Response and marathon performance groups were classified by the 33.3rd and 66.6th percentile of Δ*v*_10_ and the marathon performance time, respectively.

**Results:** Subjects allocated in the faster marathon performance group showed systematically higher training volume and higher shares of training at low intensities. Only subjects in the moderate and high response group increased their training velocity continuously along the 16 weeks of training.

**Conclusion:** We demonstrate that a combination of maximized training volumes at low intensities, a continuous increase in average running speed up to the aimed marathon velocity and high intensity runs ≤ 5 % of the overall training volume was accompanied by an improved 10 km performance which likely benefited the marathon performance as well. The study at hand proves that unsupervised workouts recorded with fitness apps can be a valuable data source for future studies in sport science.

## 1. Introduction

Finishing a marathon is a fascinating goal, especially for recreational runners. More and more people follow this dream in recent years, which is indicated by the rising number of marathon participants (Knechtle et al., 2018; Vitti et al., 2020). The motivations for people to take on this huge effort are manifold. They can be of personal (goal achievement), social (respect of peers), physical (lose weight), and psychological (becoming less anxious) manner (Zach et al., 2017). Independent of the motives behind the decision to participate in a marathon, all those runners are united in the task to prepare well by bringing their bodies in shape to run 42.2 km.

Marathon preparation techniques have been under scientific investigation for decades. Many researchers have evaluated long distance runners' training load by analyzing their training strategies with respect to volume and intensity. A high training volume has been proven to positively influence marathon performance (Hagan et al., 1987; Gordon et al., 2017). Especially recreational runners with lower training volumes can potentially increase their performance by increasing the amount of training. This was underlined by the results of Roecker et al. (1998) and Tanda (2011), who found training volume to be one of the key predictors for marathon performance in recreational runners.

In regard to the training intensity, various overviews outline advantages when training intensity is distributed in a polarized, i.e., non-uniform manner (Seiler and Tønnessen, 2009; Hydren and Cohen, 2015; Zinner, 2016; Rosenblat et al., 2019). Such concepts suggest spending certain proportions of the total training time within a low intensity (LIT) zone, a high intensity (HIT) zone, and optionally a threshold zone. In practice, training zones are either defined from a cardiopulmonary exercises test at defined percentages of the maximal oxygen uptake (V'O2max), at intensities related to ventilatory or lactate thresholds (Meyer et al., 2005) or alternatively at percentages of maximum heart rate (Seiler and Tønnessen, 2009) as well as at percentages of target marathon velocity (Billat et al., 2001; Kenneally et al., 2018).

Overall, a significant body of research provides evidence that certain physiological factors and training characteristics are systematically related to marathon performance. However, it has yet to be shown which training characteristics are the most effective in terms of an actual fitness improvement to positively influence an individual's marathon performance. In order to demonstrate whether certain training characteristics lead to higher fitness improvements, the natural variability of the individuals' responses to training has to be considered (Bouchard and Rankinen, 2001; Ross et al., 2019).

Current findings mainly result from studies with defined, recruited, and instructed cohorts. Such supervised investigations suffer from low participant numbers. In contrast, longitudinal and unsupervised activity data from large populations recorded in a natural habitat might enable sport scientists to derive more generalizable conclusions. Nowadays, millions of runners with different fitness levels track their training progress by uploading recorded data from portable sensors onto platforms like Garmin, Strava, Runtastic, etc. The challenge in working with this kind of data lies in its unsupervised nature. The data are unlabeled, which means that values of ground truth and contextual subject information for specific research questions are missing. Besides, the accuracy of the portable sensors used to acquire the data is unknown. Due to this reasons, Hicks et al. (2019) postulated that a plausibility check of the data from portable sensors is an integral part prior to its analysis. Different publications have already shown the potential of portable sensor data from fitness apps to further improve performance prediction (Altini and Amft, 2018; Berndsen et al., 2020; Emig and Peltonen, 2020), to accurately determine the critical speed of runners and to set up pacing strategies (Smyth and Muniz-Pumares, 2020) and also to individualize training plans for marathon preparation (Feely et al., 2020).

Longitudinal investigations of physical activities before a marathon appear to be a promising approach to further improve the applicability, impact, and efficiency of marathon training plans. To the best of our knowledge, there is no research which evaluated systematic differences in marathon training characteristics in relation to its response based on longitudinal data from a large unsupervised study cohort. Thus, we contribute to the state of the art in the following way:

1. We retrospectively analyze the response to training using data from portable sensors. We assess response by comparing runs of the same distance with comparable heart rates as proposed by Boullosa et al. (2020).
2. Based on the quantity of response, we define different response groups and analyze corresponding differences in total training volume and training intensity distribution within a 16 weeks training period prior to a performed marathon.
3. Respectively for each response group, we further analyze corresponding differences in total training volume and training intensity distribution between different marathon performance groups within a 16 weeks training period prior to a performed marathon.

## 2. Methods

### 2.1. Data Set

After extensive filtering (explained below) we evaluated the marathon training of 6,771 runners. We used data recorded by portable sensors such as smartphones, smartwatches, or heart rate chest straps from anonymized users of the Runtastic fitness app for the evaluation. The subjects were chosen based on the following criteria:

- one workout between 2017 and 2019 with a total distance between 41 and 43 km
- at least 16 workouts in 16 weeks leading up to the marathon
- GPS and heart rate data for each workout

We defined a range around the exact marathon distance of 42.2 km in order to include marathons of slightly different distance and inaccuracies of GPS devices used to track the marathon. Apart from distance, no additional requirements like profile or location were set for the marathon workout. The threshold of 16 workouts in the 16 weeks leading up to the marathon was empirically chosen to assure a minimum amount of data for evaluation. The data set included 5,288 male subjects (78.1%), 1,250 female subjects (18.5%), and 233 subjects of unknown sex (3.4%). The subjects' mean age was 38.5 ± 9.7 years. Body weight and height were not taken into account, because they were not available for all subjects. The GPS data (latitude, longitude) and heart rate data were sampled with different sampling rates. However, data streams of each workout were synchronized by global timestamps (UTC). GPS data was anonymized by adding a random offset to the data stream. The study is in accordance with the Declaration of Helsinki, because the local ethics committee raised no objection to its conduction due to the anonymized nature of the data.

### 2.2. Data Processing

#### 2.2.1. Extracting Overall Subject Features

For normalization purposes in later processing stages, we extracted the average marathon performance velocity *v*_*mp*_ and the maximum training heart rate *hr*_*max*_ for each subject. The average marathon performance velocity *v*_*mp*_ was determined by the duration of the marathon performance time *T*_*mp*_ for the distance between 41 and 43 km. The maximum training heart rate *hr*_*max*_ was determined to be the median of the five highest recorded heart rates over the whole training process. We decided for this approach to cope with short term outliers in the heart rate recordings.

#### 2.2.2. Feature Extraction of Individual Workouts

We computed a set of features for each of the *W* workouts leading up to a subject's marathon. The first feature obtained from the *i*-th workout (*i* ∈ {1, 2, 3, …*W*}) was the training duration *T*_*i*_. *T*_*i*_ was computed by subtracting the first from the last UTC timestamps of the GPS data. If the workout duration was longer than 90 min, we saved an indicator *I*_*T*90, *i*_, which was used further on to evaluate how many long workouts were performed:

(1) $$I_{T90,i}=\left\{ \begin{array}{l} 0\text{ if }T_{i}<90\text{ minutes}\\ 1\text{ if }T_{i}\geq 90\text{ minutes} \end{array}\right.\;.$$

For all other GPS-based features, we computed the distance and velocity over time from the GPS data. We used the great circle distance implementation of the Python package (GeoPy, 2020) to compute the distance between two consecutive GPS recordings. This resulted in a data stream of distances between two consecutive GPS-samples over time *d*_*i*_[*n*]. This data stream was used to compute the total distance of the *i*-th workout *D*_*i*_ by computing the sum over all samples. Similar to *I*_*T*90, *i*_ (Equation 1), we computed an indicator *I*_*D*15, *i*_ for workouts with distances longer than 15 km.

In order to assess training progress, we extracted the best velocity *v*_10,*i*_ for a 10 km segment within each workout (if *D*_*i*_ ≥ 10 km). For the respective 10 km segment, we also computed the average heart rate ${\bar{hr}}_{10,i}$ during the time interval.

After dividing *d*_*i*_[*n*] by the corresponding duration between two consecutive GPS timestamps $\Delta t_{i}^{gps}\left[n\right]$, we obtained a velocity data stream *v*_*i*_[*n*]. We used this data stream to compute a distribution *T*_*i*_[Ṽ] which describes the duration a subject spent in a defined velocity bin Ṽ during the *i*-th workout. To be able to define comparable velocity bins across all subjects, we normalized the velocity data stream *v*_*i*_[*n*] by the subject's marathon performance velocity *v*_*mp*_:

(2) $$\begin{array}{l} {\tilde{v}}_{i}\left[n\right]=\frac{v_{i}\left[n\right]}{v_{mp}} \end{array}$$

The velocity bins for the distribution *T*_*i*_[Ṽ] were defined from 0.54 · *v*_*mp*_ to 1.8 · *v*_*mp*_ with a bin width of 0.02. Thus, we computed the duration distribution function in the following manner:

(3) $$\begin{array}{l} T_{i}[{\tilde{V}}_{x}]=\sum_{n\in {\tilde{V}}_{x}}\Delta t_{i}^{gps}[n]\\ \text{ with }n\;\left\{ \begin{array}{l} \in {\tilde{V}}_{0}\text{ if }{\tilde{v}}_{i}[n]\leq 0.54\\ \in {\tilde{V}}_{1}\text{ if 0}.54<{\tilde{v}}_{i}[n]\leq 0.56\\ \;\vdots \\ \in {\tilde{V}}_{64}\text{ if 1}.78<{\tilde{v}}_{i}[n]\leq 1.80\\ \in {\tilde{V}}_{65}\text{ if 1}.80<{\tilde{v}}_{i}[n] \end{array}\right. \end{array}$$

For simplicity of notation, we remove the bin indicator *x* from the relative velocity bin and denote the duration distribution for different velocity bins Ṽ as *T*_*i*_[Ṽ] in the following.

The same procedure was performed for the heart rate data *hr*_*i*_[*m*]. This data stream was normalized by the subject's maximum training heart rate *hr*_*max*_. The heart rate bins were defined from 0.5 to 1 · *hr*_*max*_ with a fixed bin width of 0.02. This procedure resulted in the duration distribution for the heart rate $T_{i}\left[\tilde{HR}\right]$.

#### 2.2.3. Grouping of Workout Features in Time Frames of 4 Weeks

In order to evaluate the training progress over time, we defined training blocks of 4 weeks similar to Berndsen et al. (2020) and computed aggregated features for those training blocks. The partition of the blocks was defined based on the marathon date. Equation (4) defines the rules by which the *i*-th workout was assigned to training block *tb*:

(4) $$i\;\left\{ \begin{array}{l} \in tb_{1}\text{ if }16\text{ weeks }\leq t_{marathon}[0]-t_{i}[0]<12\text{ weeks}\\ \in tb_{2}\text{ if }12\text{ weeks }\leq t_{marathon}[0]-t_{i}[0]<8\text{ weeks}\\ \in tb_{3}\text{ if 8 weeks }\leq t_{marathon}[0]-t_{i}[0]<4\text{ weeks}\\ \in tb_{4}\text{ if 4 weeks }\leq t_{marathon}[0]-t_{i}[0]<0\text{ weeks} \end{array}\right.\text{ for }i\in \left\{1,\;2,\;3,\;...\;W\right\}$$

In this equation, *t*_*marathon*_[0] describes the first UTC timestamp of the marathon workout. For the *y*-th training block the total training time *T*_*t*_*b*__*y*__, the total training distance *D*_*t*_*b*__*y*__, the number of workouts longer than 90 min *I*_*T*90, *t*_*b*__*y*__ and further than 15 km *I*_*D*15, *t*_*b*__*y*__ could be computed by summing the values of the workouts within the training block.

(5) $$\begin{array}{l} \begin{array}{l} T_{tb_{y}}=\underset{i\in tb_{y}}{\sum }T_{i}\\ D_{tb_{y}}=\underset{i\in tb_{y}}{\sum }D_{i}\\ I_{T90,tb_{y}}=\underset{i\in tb_{y}}{\sum }I_{T90,i}\\ I_{D15,tb_{y}}=\underset{i\in tb_{y}}{\sum }I_{D15,i} \end{array} \end{array}$$

The best 10 km velocity *v*_10,*t*_*b*__*y*__ for training block *y* was chosen from all *v*_10,*i*_ of workouts in *tb*_*y*_:

(6) $$\begin{array}{l} v_{10,tb_{y}}=\underset{i\in tb_{y}}{\max}v_{10,i} \end{array}$$

**Figure A1:**
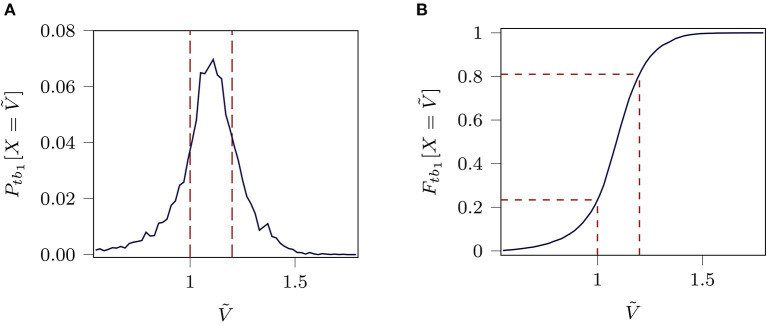
Exemplary visualization of **(A)** duration distribution curve *P*_*t*_*b*__1__[*X* = Ṽ] and **(B)** cumulative duration distribution curve *F*_*t*_*b*__1__[*X* = Ṽ] for the normalized velocity Ṽ for training block 1. The red lines indicate the barriers for the intensity zones defined based on the marathon performance velocity *v*_*mp*_.

The duration distribution curves for velocity *T*_*i*_[Ṽ] and heart rate $T_{i}\left[\tilde{HR}\right]$ were combined for the different training blocks and converted to probability distributions *P*_*t*_*b*__*y*__[*X* = Ṽ] and cumulative distributions *F*_*t*_*b*__*y*__[*X* = Ṽ] (Figure A1).

For the *y*-th training block, the duration distribution curve *T*_*t*_*b*__*y*__[Ṽ] was computed by summing the duration within the velocity bin Ṽ of all the workouts belonging to the training block:

(7) $$\begin{array}{l} T_{tb_{y}}\left[\tilde{V}\right]=\underset{i\in tb_{y}}{\sum }T_{i}\left[\tilde{V}\right]\;\forall \tilde{V}\text{ .} \end{array}$$

From *T*_*t*_*b*__*y*__[Ṽ] we computed a probability distribution *P*_*t*_*b*__*y*__[*X* = Ṽ] by dividing the time spent in the velocity bins by the total training time in *tb*_*y*_:

(8) $$\begin{array}{l} P_{tb_{y}}\left[X=\tilde{V}\right]=\frac{T_{tb_{y}}\left[\tilde{V}\right]}{\sum_{\tilde{V}}T_{tb_{y}}\left[\tilde{V}\right]}\text{.} \end{array}$$

The cumulative distribution *F*_*t*_*b*__*y*__[*X* = Ṽ] can be computed from the probability distribution by

(9) $$\begin{array}{l} F_{tb_{y}}\left[X=\tilde{V}\right]=\overset{\tilde{V}}{\underset{p=0.54}{\sum }}P_{tb_{y}}\left[X=p\right]\text{.} \end{array}$$

The same procedure was applied to the duration distribution of the heart rate to obtain the probability distribution $P_{tb_{y}}\left[X=\tilde{HR}\right]$ and cumulative distribution $F_{tb_{y}}\left[X=\tilde{HR}\right]$.

Using the probability distribution functions, we computed the normalized mean training velocity ${\bar{v}}_{tb_{y}}$ and the normalized mean heart rate ${\bar{hr}}_{tb_{y}}$ for the *y*-th training block:

(10) $$\begin{array}{l} \begin{array}{l} \;{\bar{v}}_{tb_{y}}=\underset{\tilde{V}}{\sum }\tilde{V}\cdot P_{tb_{y}}\left[X=\tilde{V}\right]\\ {\bar{hr}}_{tb_{y}}=\underset{\tilde{HR}}{\sum }\tilde{HR}\cdot P_{tb_{y}}\left[X=\tilde{HR}\right]\text{.} \end{array} \end{array}$$

We also used the distribution function of the velocity to compute the share of the workout time the subjects spent in different intensity zones. Similar to Kenneally et al. (2018) and Billat et al. (2001), we defined the zones based on the marathon velocity. The LIT zone was defined by velocities below *v*_*mp*_ and the HIT zone by velocities above 1.2 · *v*_*mp*_ (Figure A1). Using the cumulative distribution functions *F*_*t*_*b*__*y*__[*X* = Ṽ], the share of time spent in the intensity zone for *tb*_*y*_ was computed as

(11) $$\begin{array}{l} \begin{array}{l} \;{\text{LIT}}_{tb_{y}}=F_{tb_{y}}\left[X=1\right]\\ {\text{threshold}}_{tb_{y}}=F_{tb_{y}}\left[X=1.2\right]-F_{tb_{y}}\left[X=1\right]\\ \;{\text{HIT}}_{tb_{y}}=1-F_{tb_{y}}\left[X=1.2\right] \end{array}\text{.} \end{array}$$

All the computations for the training block analysis were also applied to all *W* workouts leading up to the marathon in order to obtain each subject's overall training statistics.

#### 2.2.4. Filtering Data Set

An interquartile range (IQR) filter was applied to exclude all subjects, where parameters of subjects (*T*_*mp*_, *D*, *T*, *hr*_*max*_) exceeded thresholds of 1.5·IQR below or above the lower and upper quartile.

In order to create valid response groups, we also excluded all subjects who did not achieve a minimum average heart rate of 0.8 · *hr*_*max*_ for the best 10 km runs in *tb*_1_ and *tb*_4_. 0.8 · *hr*_*max*_ was chosen to ensure sufficient cardiopulmonary effort for an individual best 10 km performance as well as a sufficient availability of data.

#### 2.2.5. Categorizing Subjects in Response and Marathon Performance Groups

Conventional metrics to assess performance improvement (i.e., V'O2max or lactate thresholds) were not available for the unsupervised data set. Therefore, we used the improvement of the 10 km velocity Δ*v*_10_ from *tb*_1_ to *tb*_4_ as a surrogate to evaluate the response of subjects to training throughout the 16 weeks before the marathon.

(12) $$\begin{array}{l} \Delta v_{10}=v_{10,tb_{4}}-v_{10,tb_{1}} \end{array}$$

A positive value for Δ*v*_10_ indicates an improvement and in turn a positive response to training and vice versa. The filter for the average heart rate stated in the data filtering section assured that those assessment runs were performed with a minimum cardiopulmonary effort. Despite the absence of conventional metrics to assess performance improvement we believe that Δ*v*_10_ is a plausible surrogate since it should reasonably reflect an improvement in endurance capacity (Roecker, 2008). Also, research has shown that the velocity of 10 km races highly correlates to marathon performances (Karp, 2007; Tanda, 2011).

**Figure 1:**
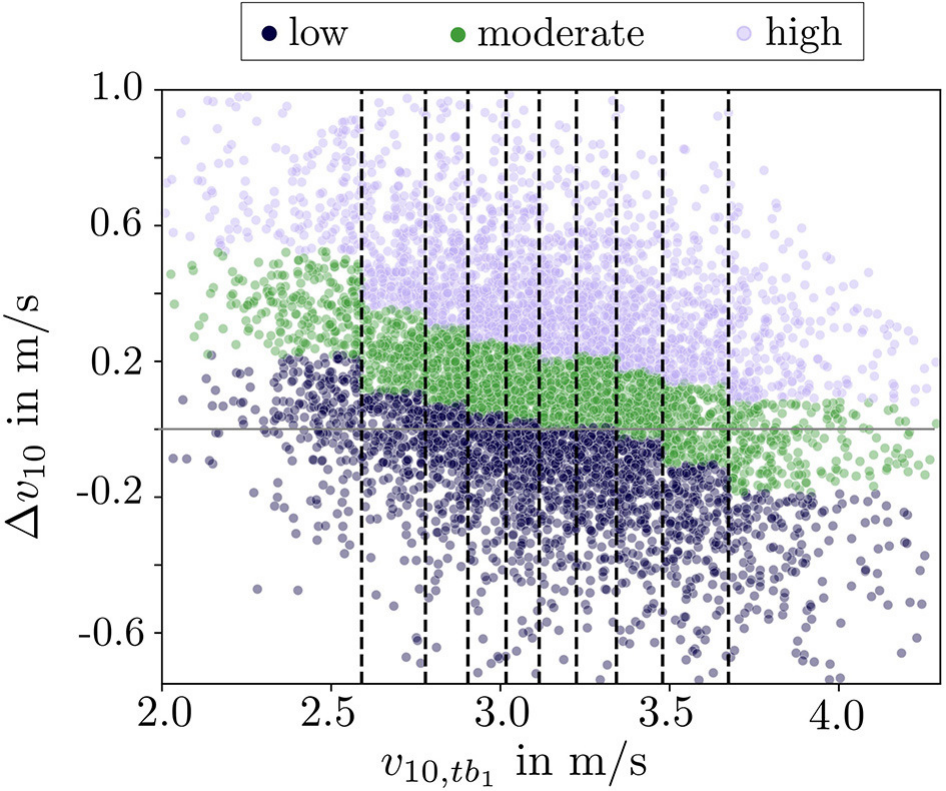
Visualization of the training response Δ*v*_10_ across ten velocity groups. Each dot represents one subject. The response categories are color coded. The vertical black lines are located at the decile values of *v*_10,*t*_*b*__1__. The horizontal gray line indicates the zero line, where subjects showed neither a positive nor negative improvement. Due to the statistical approach in the response group definition, which assured equally sized groups, the low and moderate response group also included subjects with negative Δ*v*_10_.

Δ*v*_10_ was used to categorize the subjects into three groups: high response, moderate response and low response. The borders separating the three response groups were computed at the 33.3rd and 66.6th percentile of Δ*v*_10_. We computed the percentiles for the three response groups separately on ten different *v*_10,*t*_*b*__1__ velocity groups due to decreasing improvement for subjects with higher initial *v*_10,*t*_*b*__1__ (Figure 1). The categorization of the subjects into the response groups was based on the distribution within the velocity group and not the absolute value of Δ*v*_10_. We decided for this approach to assure equally sized response groups across different performance levels.

Independent of the response group, all subjects were also categorized in three equally sized groups based on their marathon performance times using the 33.3rd and 66.6th percentile. For our data set, the 33.3rd and 66.6th percentiles referred to marathon performance times of 3 h 44' and 4 h 14', respectively. Based on those values we assigned each subject to a fast, medium and slow marathon performance group.

### 2.3. Evaluation

The evaluation consisted of three parts. Firstly, we demonstrate plausibility of the data set by reproducing known distributions and trends from literature as recommended by Hicks et al. (2019) for large unsupervised data sets. Plausibility was analyzed by plotting histograms for marathon performance times *T*_*mp*_, maximum training heart rate *hr*_*max*_, training improvement Δ*v*_10_ and a regression plot relating marathon average performance velocity *v*_*mp*_ to the best 10 km velocity *v*_10_.

Secondly, mean training velocity and mean heart rate throughout the training process were analyzed to evaluate Δ*v*_10_ as a reasonable surrogate to measure training response for each response group. Plausibility was assumed when normalized mean velocity $\Delta \bar{v}$ between *tb*_1_ and *tb*_4_ increases systematically across response groups without observing a difference in normalized mean heart rate $\Delta \bar{hr}$.

(13) $$\begin{array}{l} \begin{array}{l} \;\Delta \bar{v}={\bar{v}}_{tb_{4}}-{\bar{v}}_{tb_{1}}\\ \Delta \bar{hr}={\bar{hr}}_{tb_{4}}-{\bar{hr}}_{tb_{1}} \end{array} \end{array}$$

Differences in $\Delta \bar{v}$ and $\Delta \bar{hr}$ between response groups were analyzed using a one-way analysis of variance (ANOVA).

Lastly, means and standard deviations were derived for training parameters describing the training volume. These parameters are total distance *D*, total training duration *T*, total number of workouts *W* and number of workouts longer than 90 min *I*_*T*90_ or 15 km *I*_*D*15_ for the complete training period of 16 weeks. Additionally, means and standard deviations were derived for the training intensity parameters describing the share of time in the LIT, threshold, and HIT zone. Finally, the performance indicators relative mean velocity $\bar{v}$, best 10 km velocity *v*_10_ and relative mean heart rate $\bar{hr}$ were calculated.

Differences in the training characteristics between the response and marathon performance time groups were analyzed as follows: We computed a two-way ANOVA with the training parameter being the dependent variable and the response and marathon groups being the independent variables. We excluded *W*, *I*_*T*90_ and *I*_*D*15_ of the ANOVAs, because the values of those training parameters were not continuous. For the intensity parameters LIT, threshold and HIT, we analyzed differences in the training process over time by computing repeated measure ANOVAs for the three training zones over the four training blocks. For all ANOVAs, we report partial η^2^ effect sizes if the *p*-values showed statistical significance with a significance level of α < 0.05. All statistical tests in this work were conducted using the Python package Pingouin (Vallat, 2018).

## 3. Results

### 3.1. Plausibility of the Data Set

**Figure 2:**
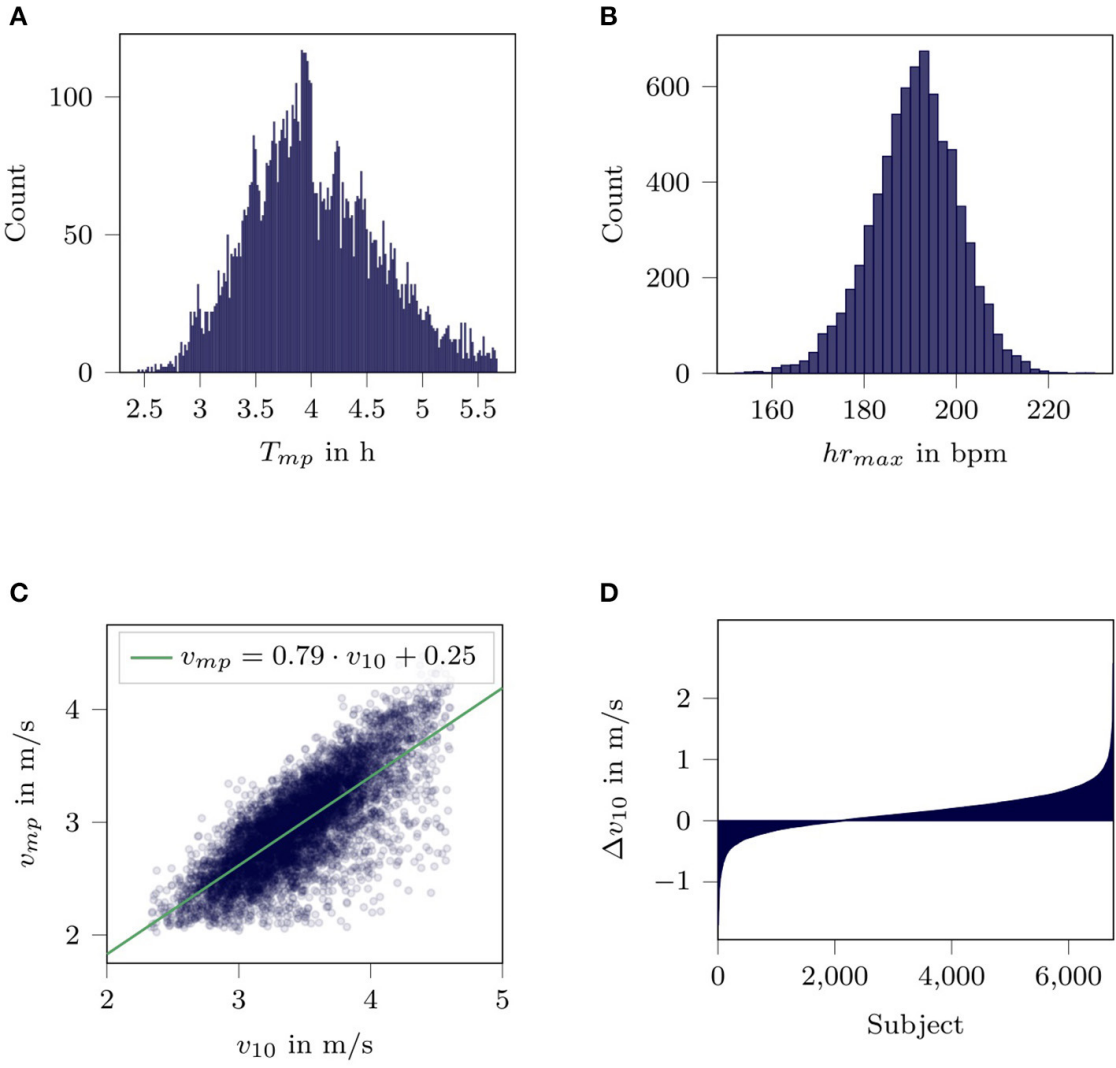
Validation of data set. **(A)** Distribution of marathon performance time *T*_*mp*_. **(B)** Distribution of maximum training heart rate *hr*_*max*_. **(C)** Visualization of the correlation between best 10 km velocity *v*_10_ in the complete training period and marathon performance velocity *v*_*mp*_. The blue dots indicate the individual subjects, the green line the linear regression function. **(D)** Adaptive potential for improvement of best 10 km velocity Δ*v*_10_.

Figure 2 depicts the results for the plausibility of the data set. The distribution of the marathon performance reached from 2.5 up to 6 h. We noticed distinct peaks at the full and half hour marks (Figure 2A). The histogram of the maximum training heart rate shows normally distributed values between 160 and 220 bpm (Figure 2B). A high correlation (Pearson's *r* = 0.77) was found between marathon average velocity and the overall best average 10 km velocity detected within the 16 weeks leading up to the marathon (Figure 2C). Lastly, a sorted distribution of Δ*v*_10_ is presented in Figure 2D. Values of Δ*v*_10_ ranged between −1 and 2 m/s indicating a negative or no improvement in less than a third of the population.

### 3.2. Evaluation of Response Groups

**Figure 3:**
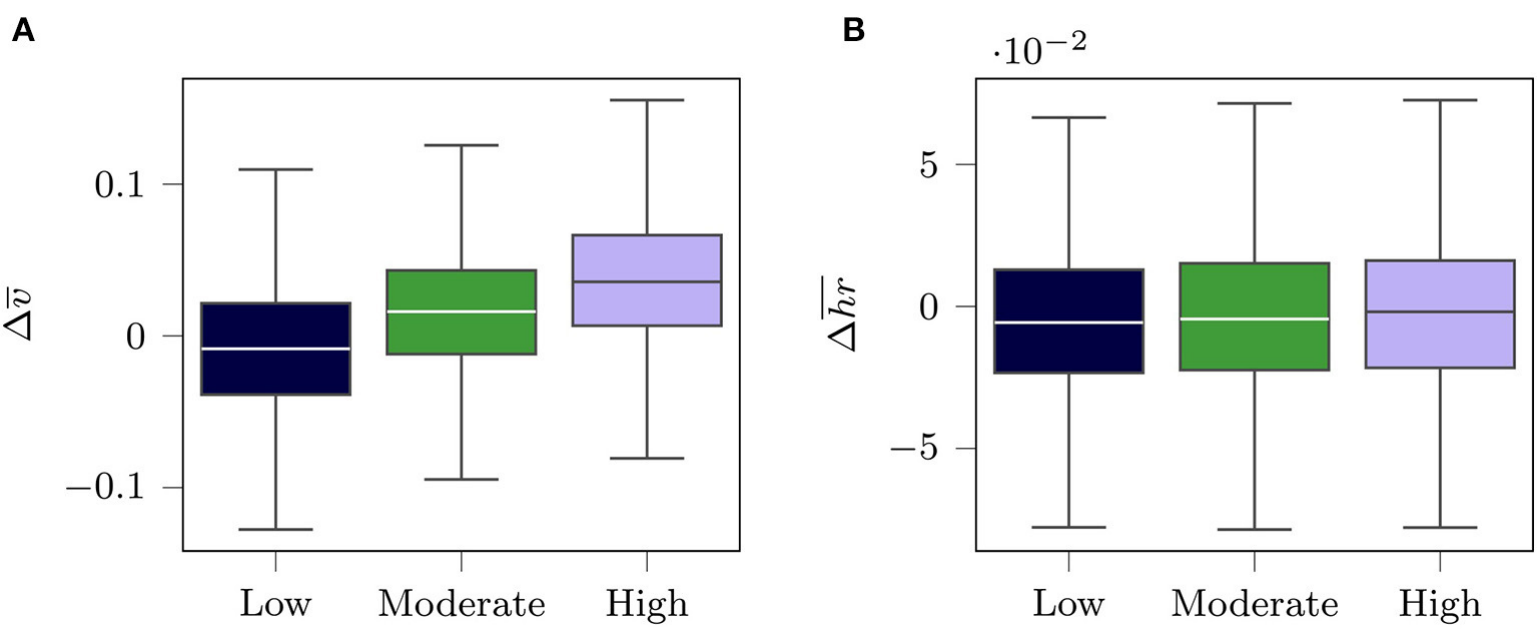
Visualization of the difference between *tb*_4_ and *tb*_1_ of **(A)** the normalized mean velocity and **(B)** the normalized mean heart rate for all subjects in the three response groups. The velocity values were normalized by the marathon performance velocity *v*_*mp*_ and the heart rate by the maximum training heart rate *hr*_*max*_.

Figure 3 depicts the verification of the response group definition. Subjects in the high response group, who showed the highest improvements in Δ*v*_10_, also showed the highest improvement in $\Delta \bar{v}$, while slightly decreasing their mean heart rate. We found a large effect size for the differences of $\Delta \bar{v}$ (η^2^ = 0.136) and a small effect sizes for difference of $\Delta \bar{hr}$ (η^2^ = 0.001) between the response groups.

### 3.3. Evaluation of Training Characteristics

Table 1 lists the mean values and standard deviations of the training parameters for subjects in the different response and marathon performance groups over all 16 weeks before the marathon. Besides, the effect sizes of the two-way ANOVA (response group, marathon performance group) for the main effects are reported in case of statistical significance (α < 0.05). We did not report effect sizes for the interaction effects, because they were not statistically significant. The results show small effect sizes for the differences between the response groups and higher effect sizes for the differences between the marathon performance groups. Our approach to categorizing subjects into response groups and marathon performance groups yielded a higher number of subjects with a fast marathon performance time in the high response group and in contrast a higher number of subjects with a slow marathon performance time in the low response group.

**Table 1:** Mean and standard deviation of the training parameters for the 16 week training process.

**Parameter**	**$\eta_{resp}^{2}$**	**$\eta_{mp}^{2}$**	**Marathon group**	**Low**	**Moderate**	**High**
*T* [h]	–	0.037	Slow	52.8, 18.7	52.5, 17.8	53.1, 19.7
			Medium	55.9, 19.3	56.1, 17.4	56.8, 18.8
			Fast	61.4, 21.3	62.3, 19.1	63.0, 19.3
*D* [km]	0.002	0.132	Slow	472.8, 164.5	482.9, 157.8	495.3, 185.9
			Medium	547.1, 178.8	558.3, 167.0	559.4, 175.9
			Fast	641.9, 213.9	671.6, 197.6	677.8, 206.7
$\bar{hr}$ [%]	0.004	0.030	Slow	0.81, 0.04	0.81, 0.03	0.80, 0.03
			Medium	0.80, 0.03	0.80, 0.03	0.80, 0.03
			Fast	0.78, 0.04	0.80, 0.03	0.79, 0.04
$\bar{v}$ [%]	–	0.360	Slow	1.09, 0.08	1.09, 0.09	1.10, 0.10
			Medium	1.01, 0.06	1.01, 0.06	1.01, 0.07
			Fast	0.96, 0.06	0.96, 0.06	0.95, 0.06
v_10_ [m/s]	0.050	0.429	Slow	3.05, 0.32	3.1, 0.27	3.33, 0.31
			Medium	3.40, 0.29	3.37, 0.24	3.49, 0.26
			Fast	3.78, 0.31	3.73, 0.30	3.87, 0.29
LIT [%]	0.003	0.363	slow	0.29, 0.17	0.28, 0.16	0.28, 0.18
			Medium	0.47, 0.19	0.47, 0.19	0.49, 0.19
			Fast	0.63, 0.18	0.62, 0.18	0.66, 0.17
thr. [%]	0.012	0.151	Slow	0.47, 0.14	0.47, 0.14	0.43, 0.14
			Medium	0.43, 0.14	0.43, 0.15	0.40, 0.14
			Fast	0.31, 0.15	0.33, 0.16	0.29, 0.14
HIT [%]	0.002	0.296	Slow	0.24, 0.18	0.24, 0.19	0.30, 0.21
			Medium	0.10, 0.09	0.10, 0.09	0.11, 0.09
			Fast	0.06, 0.06	0.06, 0.04	0.05, 0.05
W	–	–	Slow	40.7, 13.7	41.5, 14.1	41.8, 14.8
			Medium	44.2, 14.7	44.9, 13.6	46.1, 14.9
			Fast	51.4, 18.7	52.7, 16.4	53.2, 17.5
I_*T*90_	–	–	Slow	10.8, 5.4	10.5, 5.1	10.6, 5.5
			Medium	11.3, 5.4	10.9, 5.2	11.1, 5.3
			Fast	11.7, 6.1	11.4, 5.6	11.5, 5.5
I_*D*15_	–	–	Slow	9.0, 4.8	9.3, 4.6	9.6, 5.6
			Medium	11.0, 5.3	11.1, 5.3	10.9, 5.1
			Fast	12.9, 6.5	13.9, 6.5	14.1, 6.4
Subjects	–	–	Slow	1121	744	394
			Medium	709	823	723
			Fast	429	685	1143

*The statistical values are reported for the different response groups and the different marathon time categories. T indicates the total training duration, D the total distance, $\bar{hr}$ the relative mean heart rate $\bar{v}$ the relative mean velocity, v_10_ the best 10 km velocity, LIT the share of time in the LIT zone, threshold the share of time in the threshold zone, HIT the share of time in the HIT zone, W the number of workouts, I_T90_ the number of workouts longer than 90 min and I_D15_ the number of workouts with a higher than 15 km of the 16 week training process. For all parameters except W, I_T90_, and I_D15_, we report the effect sizes $\eta_{resp}^{2}$ (response groups) and $\eta_{mp}^{2}$ (marathon performance groups) of the main effect of the two-way ANOVA if the p-value was below a significance level α = 0.05. The last row of the table lists the number of subjects in each group*.

**Figure 4:**
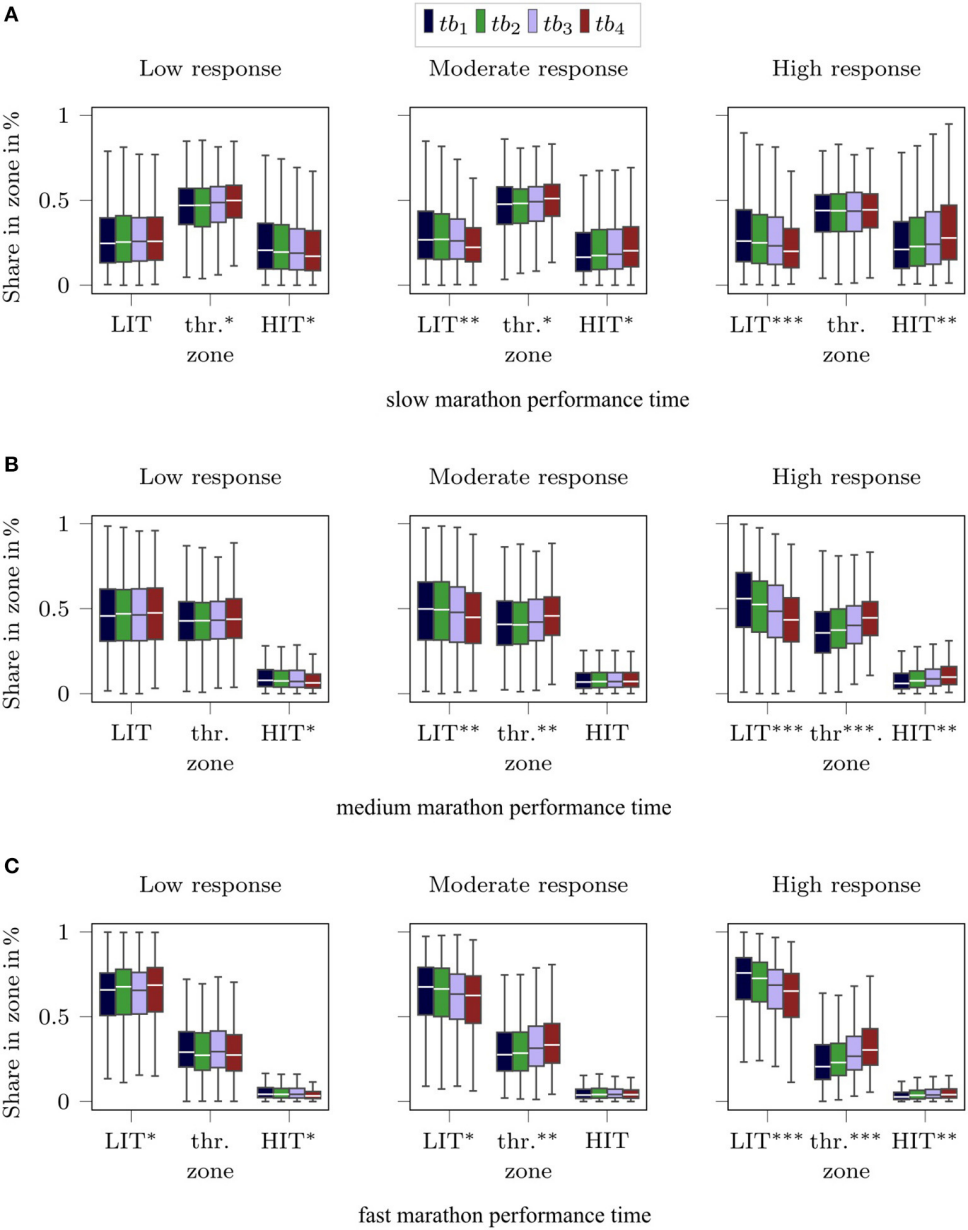
Visualization of the share of time spent in the intensity zones for **(A)** slow marathon performances, **(B)** medium marathon performances, and **(C)** the fast marathon performances. For each marathon performance group, we provide three plots showing the share of time spent in the three zones for the low response, moderate response, and high response group. The individual boxes in each plot visualize the IQR within the training blocks. The black horizontal lines within the boxes indicate the median. The whiskers extend to 1.5·IQR. We computed repeated measure ANOVAs for each response and marathon performance category for each intensity zone. The asterisks indicate the effect size of the results: *0.01 ≤ η^2^ <0.05, **0.05 ≤ η^2^ <0.12, ***η^2^≥0.12.

Figure 4 depicts the share of time spent in the three intensity zones during the four training blocks for the subjects in the different response and marathon performance groups. It shows differences in the intensity distributions between slow, medium, and fast runners. We observe an increasing share of training time in the LIT zone from the slow to the fast marathon group. Within the marathon performance group, the overall amount of time spent in the individual zones remains constant.

**Figure A2:**
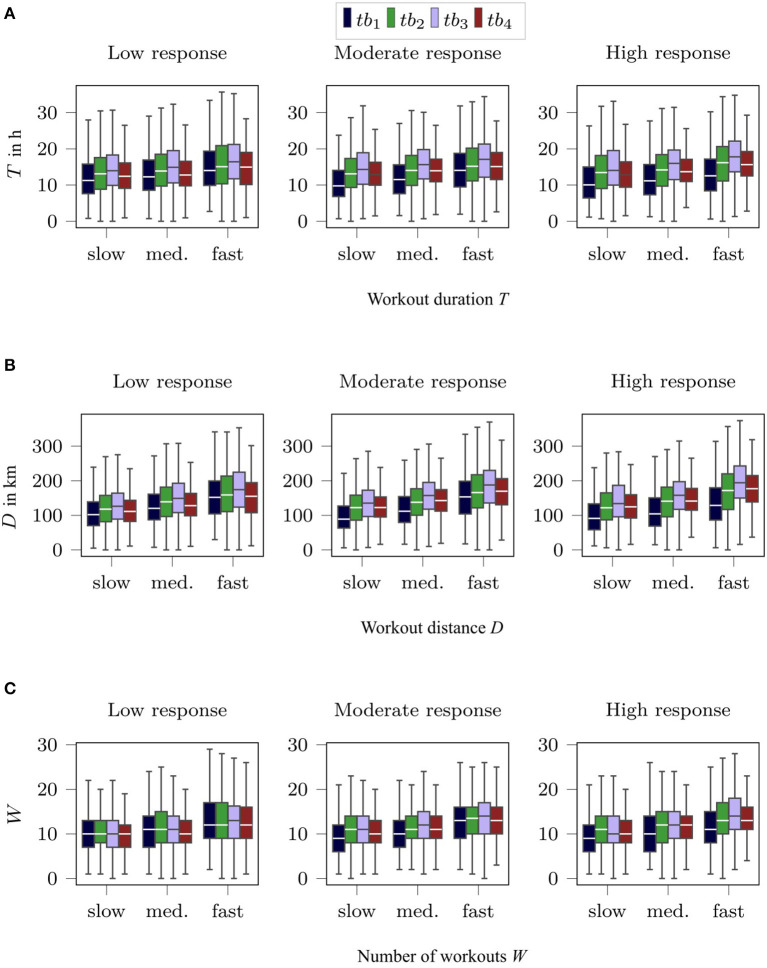
Visualization of training parameters over training blocks. **(A)** Workout duration *T*, **(B)** workout distance *D*, **(C)** number of workouts *W*.

However, differences in time spent in the intensity zones between the four training blocks were found. Especially subjects allocated in the high response group decreased the time in the LIT zone throughout the training process, while increasing the share of time in the threshold and HIT zone. This is underlined by the results of the repeated-measures ANOVA for each combination of response and marathon time category in each zone over the training blocks. In Figure 4, the effect sizes of the statistical tests are indicated by asterisks. Subjects allocated in the high response group revealed the highest effect sizes for differences of time spent in the three intensity zones between the four training blocks. Differences in training volume parameters between the four training blocks were also analyzed but did not show any significant differences between the three response groups (Figure A2).

## 4. Discussion

In this study, we performed a large-scale retrospective data analysis of runners' training in the 16 weeks leading up to a marathon. The aim of the analysis was to evaluate differences in training characteristics between different response and marathon performance groups. The data used for the analysis were originated by members of the Runtastic fitness app who used portable sensors to track their training progress. From the initial data set of 14,773 marathon finishers only 6,771 subjects remained after applying filters to improve data quality. In particular, the filter ensuring that the subjects performed the 10 km effort in *tb*_1_ and *tb*_4_ with an average heart rate > 0.8 · *hr*_*max*_ reduced the number of subjects by 6,845. We believe that this drastic reduction of more than 50 % was necessary to ensure a conclusive analysis.

### 4.1. Plausibility of the Data Set

By reproducing known values and trends from literature as suggested by Hicks et al. (2019), we could verify that our data set can be used for the analysis of differences in training leading up to a marathon. The distribution of marathon performance times is similar to the one presented by Allen et al. (2017), including the peaks at the full and half hour marks. Thus, even though the data query only required a workout between 41 and 43 km, the marathon performance times indicate that the workouts were actual marathon races. This assumption is supported by the fact, that 98.6% of the marathon workouts were performed on the weekend. The distribution of the maximum training heart rate *hr*_*max*_ shows realistic results similar to data observed by others (Roecker et al., 2002; Sarzynski et al., 2013), who determined maximum heart rates using laboratory exercise tests. Thus, we believe that the maximum training heart rate *hr*_*max*_ also reflects the actual maximum heart rate well.

Strong correlations between average marathon velocity and average 10 km velocity have been reported by others (Karp, 2007; Tanda, 2011) and are verified by our data. The sorted values for Δ*v*_10_ show a heterogeneity in response to training. In comparison to the findings from Bouchard and Rankinen (2001), the portion of the population who showed a negative or no improvement in our investigation was higher. We believe that the higher portion was due to the unsupervised nature of the data as well as the low threshold of > 0.8 · *hr*_*max*_ we set to verify the best 10 km performances. However, increasing the threshold of *hr*_*max*_ to elevate the cardiopulmonary effort for the best 10 km velocities did not change the proportion of training responses.

In comparison to supervised studies from Gordon et al. (2017) and Hagan et al. (1987), we observed lower weekly mean values in number of workouts, total training duration, and total distance. However, reduced mean values in training volume have also been shown in other unsupervised investigations (Leyk et al., 2009; Smyth and Muniz-Pumares, 2020). Lower training volumes might be caused by the heterogeneous nature of the larger data set itself.

### 4.2. Evaluation of Response Groups

We introduced an approach to assess physical fitness based on the best 10 km velocity *v*_10_ that was accompanied by a heart rate > 0.8 · *hr*_*max*_. We classified three equally large response groups based on observed changes in the average 10 km velocity in *tb*_1_ and *tb*_4_. The idea of frequently monitoring typical training sessions to evaluate the response to training has already been proposed by Boullosa et al. (2020) and appears very practical. This is especially the case when data from recreational runners are analyzed, where laboratory fitness assessments are usually not part of the individual training routine. The 10 km velocity was chosen due to its high correlation to the marathon average velocity (Karp, 2007; Tanda, 2011). Therefore, we assume that an improvement of *v*_10_ should also positively influence the marathon performance velocity *v*_*mp*_.

In addition, a systematic increase in mean normalized running velocity was found when comparing the three response groups from low to high response while no systematical differences in mean normalized heart rate were present. This provides further evidence that in general Δ*v*_10_ likely reflects an improved physical fitness, even though the cause for the improvement may vary between individuals (e.g., improvement due to following a specific training structure with fast runs at the end of the 16 weeks training period). Ultimately, the fact that there were more subjects with a fast marathon performance time allocated in the high response group gives final confirmation that our approach to classify the three response groups based on Δ*v*_10_ is reasonable.

### 4.3. Evaluation of Training Characteristics

The evaluation of training characteristics between marathon performance groups revealed differences with medium to large effect sizes. The mean values of all parameters describing the training volume (*D*, *T*, *W*, *I*_*T*90_, *I*_*D*15_) are systematically higher for the faster marathon performance time group. Similar relationships were also reported elsewhere (Hagan et al., 1987; Tanda, 2011; Gordon et al., 2017). In accordance with others, our results also demonstrate that polarized training with maximized volumes below the aimed marathon velocity in the LIT zone yield better marathon performances (Seiler and Tønnessen, 2009). While slow marathon performance times were associated with the largest shares of training time in the threshold zone, fast marathon finishers spend on average more than 60% of their training time in the LIT zone below their average marathon velocity. The larger shares in the threshold zone for the medium and slow marathon groups might be due to the fact that recreational runners cannot control intensity well and tend to run too fast even for prescribed training plans (Foster et al., 2001).

The mean training parameters in Table 1 showed no differences between the response groups (all η^2^ < 0.012). This implicates that high training volumes do not influence the response to training in general. This should be of interest to novice runners, who are at higher risk to be injured from too high training loads (Buist et al., 2010; Videbæk et al., 2015). Nevertheless, the response groups differed regarding the shares of time spent in the three intensity zones throughout the four consecutive training blocks. Independent of the marathon performance time, we observed strong effect sizes for decreasing duration in the LIT zone across the four training blocks for subjects in the high response group. While this observation of course is partly a result of our definition of the response groups, the analysis demonstrates that those subjects who started to train at very low velocities and continuously increased their training velocity up to the actual marathon velocity throughout the 16 weeks responded to the highest extent, leading up to at least an average (<4 h 14') or even a fast marathon time (<3 h 44′).

### 4.4. Limitations

Despite all the filters applied to improve data quality, a study with unsupervised data from fitness apps cannot be as controlled as a supervised study. For our investigation, we are not able to guarantee that all subjects logged and uploaded all physical activities which could have influenced their 10 km or marathon performance. Contextual information affecting the performance of runners like humidity and temperature during a workout or an injury of a runner were not available. The results are also influenced by the varying accuracy of the different portable sensors recreational runners use to track their workouts. Running velocity was not adjusted to the elevation profile of the running route, which neglects the impact of inclines and declines to training load. Additionally, phenomenons like “hitting the wall” during a marathon (Buman et al., 2008) were not controlled for, which might cause subjects to be classified in a worse marathon performance category despite a good training process. We acknowledge that these limitations might affect the results of individuals in our analysis. However, we believe that the number of those individuals is low compared to the overall number of subjects and that the effect for most of the limitations are equally distributed over the response and marathon performance groups. Thus, differences between or within groups should not be affected. Nevertheless, a detailed analysis of the influence of those limiting factors on the response to training and the marathon performance shall be conducted in future work.

## 5. Conclusion

In this work, we retrospectively analyzed 16 weeks of training for 6,771 marathon finishers. We showed that unsupervised data recorded by portable sensors are suitable for performing such an analysis by reproducing known trends and values from literature. Our analysis demonstrated that a combination of maximized training volume at velocities below an individual's marathon velocity, a continuous increase in average running velocity along the complete training period up to final average marathon velocity and high velocity runs (> 1.2 · *v*_*mp*_) not accounting for more than 5% of the overall training volume was associated with a higher Δ*v*_10_ which likely benefited the marathon performance as well. We also demonstrated that a high training volume does not generally influence the response to training.

The large variances in both the training characteristics and the corresponding responses indicate that the most effective training plan for an individual has yet to be developed. However, coaches and athletes also have to acknowledge that, even with the best and most effective training plan, the potential to improve performance is limited and partially genetically determined.

This study also showed that data recorded by portable sensors and stored on various fitness platforms are an extremely valuable source for investigating different training regimes retrospectively on large sample sizes. Especially for longitudinal investigations, the limitation of low sample sizes can be overcome. This might enable sport scientists and training physiologists to draw more generalizable conclusions in the future.

## Data Availability Statement

The data set originated from the Runtastic data base. We agreed to not publish the raw data, but only aggregated results. Requests to access the aggregated results should be directed to markus.zrenner@fau.de.

## Ethics Statement

The studies involving human participants were reviewed and approved by Ethics committee FAU Erlangen-Nürnberg. Written informed consent from the participants' legal guardian/next of kin was not required to participate in this study in accordance with the national legislation and the institutional requirements.

## Author Contributions

MZ designed the study, implemented the methodology, interpreted the results, and wrote the manuscript. CH interpreted the results, wrote, and reviewed the manuscript. BD designed the study and reviewed the manuscript. SD exported and anonymized the data set and reviewed the manuscript. KR interpreted the results and reviewed the manuscript. BE designed the study, interpreted the results, and reviewed the manuscript. All authors have read and approved the final version of the manuscript and agree with the order of presentation of the authors.

## Conflict of Interest

CH and BD were employed by the adidas AG. SD was employed by the Runtastic GmbH. The remaining authors declare that the research was conducted in the absence of any commercial or financial relationships that could be construed as a potential conflict of interest.
